# Interplay Between Skin Microbiota Dysbiosis and the Host Immune System in Psoriasis: Potential Pathogenesis

**DOI:** 10.3389/fimmu.2021.764384

**Published:** 2021-10-18

**Authors:** Xiaoqian Liang, Caixin Ou, Jiayi Zhuang, Jinsheng Li, Fangfei Zhang, Yuanqiu Zhong, Yongfeng Chen

**Affiliations:** Department of Dermatology, Dermatology Hospital of Southern Medical University, Guangzhou, China

**Keywords:** psoriasis, skin microbiota, immunology, pathogenesis, Th17, inflammation

## Abstract

Psoriasis is a multifactorial immune-mediated disease. The highly effective and eligible treatment for psoriasis is limited, for its specific pathogenesis is incompletely elucidated. Skin microbiota is a research hotspot in the pathogenesis of immune-mediated inflammatory skin diseases nowadays, and it may have significant involvement in the provocation or exacerbation of psoriasis with broadly applicable prospects. It is postulated that skin microbiota alternation may interplay with innate immunity such as antimicrobial peptides and Toll-like receptors to stimulate T-cell populations, resulting in immune cascade responses and ultimately psoriasis. Achieving a thorough understanding of its underlying pathogenesis is crucial. Herein, we discuss the potential immunopathogenesis of psoriasis from the aspect of skin microbiota in an attempt to yield insights for novel therapeutic and preventive modalities for psoriasis.

## 1 Introduction

Psoriasis is a chronic systemic inflammatory disease contributed by genetic, immunological, and environmental factors, with a prevalence rate of 2%–4% of the worldwide population (1). Thus far, psoriasis has no known cure, and innovative biological therapies are of great prospects. Emerging evidence supports the vital role of gut microbiota in psoriasis, and the relevant products such as probiotics have facilitated its treatment improvement, with an encouraging reduction in Psoriasis Area and Severity Index (PASI) and lower risk of relapse during follow-up (2, 3). In view of psoriasis being a skin disease, skin microbiota alternation may act more significantly to trigger or exacerbate psoriasis. Current research has put emphasis on the role of skin microbiome in immune-mediated inflammatory skin diseases, including atopic dermatitis, acne vulgaris, vitiligo, and systemic lupus erythematosus (4–8), yet there are certain blank regions in psoriasis. Even though the causal link between skin microbiome and psoriasis remains elusive, evolving knowledge supports its potential role interplaying on T helper cell 17 (Th17) in psoriatic patients, acting on inflammatory cells and cytokine pathways to induce immunity disorder on skin, confirmed by detection of plasma metabolism and bacterial metabolites (9). Furthermore, patients with psoriasis have higher risks of associated comorbidities, such as psoriasis arthritis (PSA), inflammatory bowel disease, periodontitis, cardiometabolic dysfunction, depression, and metabolic syndrome, which may be due to concurrent susceptible locus and aberrant impaired tolerance in response to microbiota (10–14). With the update of whole-genome shotgun (WGS) metagenomic sequencing (15), the assessment accuracy of bacterial species, community diversity, and gene prediction has been greatly optimized in trials. In the forthcoming period, specific skin microbiome signature might be a promising concept for the diagnostic, therapeutic, and preventive strategies for psoriasis. Novel therapeutic modalities that target or restore the altered skin microbiota are valuable adjuncts for the management of psoriasis and its comorbidities. This article describes the potential pathogenesis of psoriasis concerning how skin microbiota interplay with the host immune system, aiming at providing new insights regarding the immunopathogenesis of psoriasis. A good knowledge about their intrinsic and comprehensive interplay is certainly beneficial to form the basis of innovative treatment selection and may help revolutionize an unprecedented era of biologic therapies for psoriatic patients.

### 1.1 The Widely Accepted Immunopathogenesis of Psoriasis

The predominant pathogenesis of psoriasis involves a cross-talk between innate and adaptive immunity through interleukin (IL)-23/Th17 axis and immune responses on resident skin cells (16). It is well recognized that overactive responses of IL-17-producing dermal T cells, namely, Th17 cells, play a significant role in triggering psoriasis (17). Stimulated by antimicrobial peptides (AMPs) such as LL-37 produced by epidermis, dendritic cells (DCs) release IL-12, IL-23, etc., to expand the differentiated Th17 cell populations, in which IL-23 acts significantly (18, 19). Alongside Th1 cells, and Th22 cells, Th17 subsets produce pro-inflammatory mediators such as IL-17A, 17F, tumor necrosis factor (TNF)-α, interferon (IFN)-α, and IL-22, leading to hyperproliferation of keratinocytes and immunocyte infiltration on skin to amplify psoriatic inflammation (20, 21). Of note, there is a positive feedback loop that the activated and proliferative keratinocytes subsequently release richer AMPs and cytokines such as IL-1, IL-24, CCL20, and CXCL1–3, serving as chemoattractants to facilitate leukocyte recruitment, angiogenesis, and further keratinocyte proliferation in psoriatic skin (22).

Growing experience has revealed that IL-17 could downregulate filaggrin and genes associated with cellular adhesion to induce skin barrier disruption (23), thereby eliciting a hypoxia environment and the upregulated expression of vascular endothelial growth factor (VEGF) as part of the pathological basis of psoriasis (24), which is in agreement with the theory that psoriasis could be triggered in the context of reactive oxygen species (ROS) production and a decrease in antioxidant activity (25). On the other hand, markedly higher IL- 9R and IL-9 expression was detected in psoriatic skin lesions. IL-9 would incur micrangium angiogenesis and Th17-associated inflammation by stimulating angiogenic markers (VEGF and CD31) and promote secretion of IL-17, IL-13, IFN-γ, and TNF-α (26). By and large, a psoriasis lesion is provoked by either exogenous skin disruption or endogenous infiltration of activated immunocytes (22). On the basis of preliminary studies indicating the key regulatory function of nuclear factor-κB (NF-κB) in TNF production, cell death, immune infiltration, and hyperkeratosis, NF-κB and mitogen-activated protein kinase (MAPK) signaling pathways have been identified to organize psoriatic skin in a Th17-associated manner (27, 28).

### 1.2 Normal Skin Microbial Community

As the powerful protective barrier, skin is home to diverse immune functions and trillions of microorganisms. Generally, skin microbiota consists of resident and transient flora, along with dynamic characteristics across space and time (29), shouldering their responsibility for host homeostasis on skin principally through effector T cells and DCs (30).

Since microbiota composition is altered in accordance with genetics, site, sampling techniques, etc., there is no uniform standard of healthy skin microbiome. Notwithstanding, 90% of individuals have a “core” commensal microbiome encoding unique products, with remarkable ability to calibrate both innate and adaptive immunity, further completed by Human Microbiome Project (31, 32). On healthy skin, the predominant four phyla are as follows: Actinobacteria (51.8%), Firmicutes (24.4%), Proteobacteria (16.5%), and Bacteroidetes (6.3%); and the three most prevalent genera are *Corynebacterium* (22.8%; Actinobacteria), *Propionibacterium* (23.0%; Actinobacteria), and *Staphylococcus* (16.8%; Firmicutes) (33). With regard to fungi, *Malassezia* is the predominant flora (34).

### 1.3 Psoriasis-Associated Microbial Community

Epithelial immune microenvironment regulates the pathogenic inflammatory loop in psoriasis, in which keratinocytes serve as amplifiers and microorganisms as the primary trigger (35). Cutaneous innate immune defenses, including Toll-like receptors (TLRs), pattern recognition receptors, proteoglycan recognition proteins (PGRPs), and antimicrobial peptides (AMPs) have been implicated as potential contributors to psoriasis *via* Th1/Th17 cell populations in response to cutaneous microbiota (36). TLRs and PGRPs, also called pathogen-recognition receptors (PRRs), are capable of recognizing microbiota and thus altering innate and adaptive immune responses (37). While AMPs, such as LL37, Human β-defensins (HBD), and human S100A7, can not only resist pathogens but also elicit chemotaxis, angiogenesis, and keratinocyte proliferation (22). It has been demonstrated that IL-23 upregulated Human β-defensins (HBD)-2 expression to stimulate keratinocyte proliferation and cytokine production along with Th17 cell expansion, probably involved in the pathogenesis of psoriasis (38).

**Figure 1:**
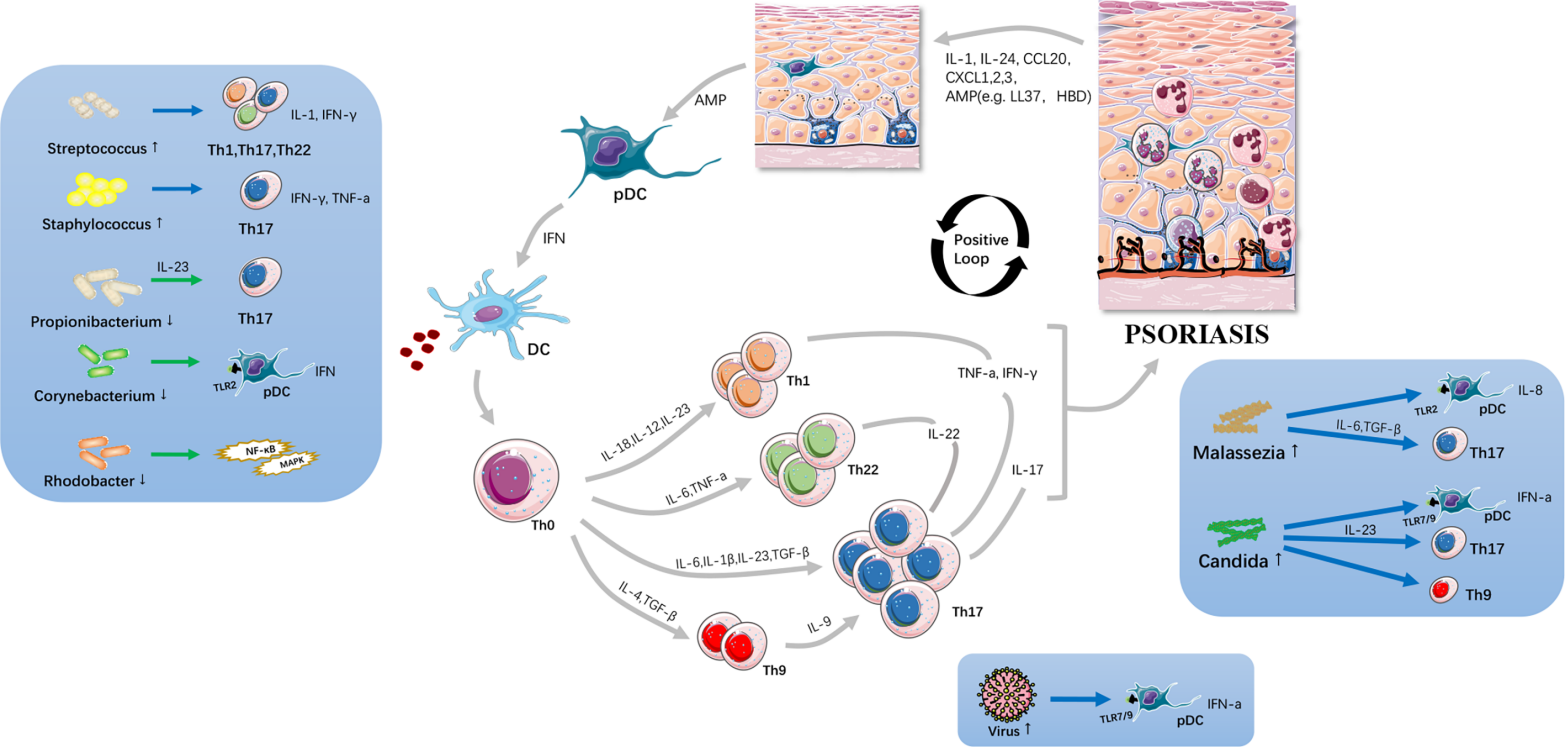
Potential immunopathogenesis of psoriasis. The altered skin microbiota cause skin barrier disruption and act on innate immune system including Toll-like receptors etc.; and, subsequently drive Th17-associated inflammatory cascades. Large amounts of cytokines are secreted, resulting in immunocyte infiltration, angiogenesis and keratinocyte proliferation thus resulting in initiation and progression of psoriasis. Skin microbiota is viewed as a significant trigger and exacerbator in psoriatic inflammation loop.

Ongoing efforts are further completely establishing the pathogenesis of psoriasis and yielding insights for more effective treatment paradigms. Herein, we summarize multiple clinical trials regarding the altered skin microbiota in psoriasis (**Tables 1** **–** **3**), shedding light on how skin microbiota influence host innate and adaptive immunity under the inflammatory condition in an effort to unveil the underlying causative association between psoriasis and skin microbiota dysbiosis (**Figure 1**). As a whole, psoriatic lesions trend to decreased taxonomic and species level diversity in terms of both richness and evenness, whereas with greater intragroup variability. Studies proposed that the altered skin microbiota may promote their translocation into the bloodstream or shed some cell components as inflammagens, thereby driving the systemic inflammation in the host. The commensal skin microbes are reported with weak relevance with disease-related transcripts (50).

**Table 1:** Summary of published clinical trials involving skin bacterial microbiota in psoriasis.

Study	Psoriasis Patients			Healthy Controls			Psoriasis type	Psoriasis severity	Sampling	Method
	Number	Mean or Median Age	Sex ratio (M:F)	Number	Mean or Median Age	Sex ratio (M:F)				
(9)	32	38.16 (17–74)	–	29	35.53 (23–54)	–	Severe psoriasis (PASI score ≥12)	PASI: 38.96 ± 2.64 BSA: 24.85 ± 2.82 PGA: 4.41 ± 0.13	Swab	16S rRNA (V3–V4)
(39)	28	42.3 ± 14.1	11:7	26	43.6 ± 15.1	10:16	Plaque psoriasis	PASI: 11.1 ± 8.9	Swab	16S rRNA (V1–V3)
(40)	8	–	–	8	–	–	–	–	Swab	16S rRNA
(41)	1	50 ± 3 (all subjects)	1:0	1	50 ± 3 (all subjects)	1:0	–	PASI: 20.0	Curettage	16S rRNA (V2–4–8, V3–6,7–9)
(42)	51	49.1 ± 16.4	39:12	37	–	–	Chronic plaque psoriasis	PASI: 8.7 ± 10.1 BSA: 9.4 ± 13.9 PGA: 6.6 ± 6.9	Swab	16S rRNA (V1–V3)
(43)	10	24–60	5:5	12	34–62	7:5	Chronic plaque psoriasis	–	Biopsy	16S rRNA (V3–V4)
(44)	6	–	3:3	–	–	–	–	BSA: 12 ± 5.7	Swab	16S rDNA

PASI, Psoriasis Area and Severity Index; BSA, Body Surface Area score; PGA, Psoriasis Global Assessment score.

**Table 2:** Summary of bacterial diversity and taxonomic characteristics on psoriatic skin in clinical trials.

Study	Diversity	Relative Abundance			
		Phyla level	Family level	Genus level	Species level
(9)	α: Not significant	–	–	*Lactobacillus*: L > H (p < 0.001) *Luteimonas*: L > H (p = 0.05) *Thermomonas*: L>H (p = 0.02) *Vibrio*: L > H (p < 0.05)	–
(39)	α: L > U > H (p = 0.005) β: Not significant	*Actinobacteria*: H > U > L *Proteobacteria*: L > U > H	–	*Propionibacterium*: H > U > L	*Propionibacterium acnes*: H > U > L (p = 0.0002) *Propionibacterium granulosum*: H > U > L (p = 0.014) *Staphylococcus sciuri*: U > L > H (p = 0.032) *Staphylococcus aureus*: L > U > H (p = 0.007) *Staphylococcus pettenkoferi*: L > U > H (p = 0.012) *Staphylococcus epidermidis*: H > U > L (not significant)
(40)	α: H > L (p = 0.04)	*Actinobacteria*: H > L (p = 0.0001) *Firmicutes*: L > H (p = 0.009)	–	*Alloiococcus*: L > H (p = 0.01), U > H (p = 0.003) *Aerococcus*: L > H (p = 0.01) *Propionibacterium*: H > L (p = 0.08) *Gallicola*: L > H (p = 0.09), L > U (p = 0.04)	*Acinetobacter* spp.: L ≈ U > H *Staphylococcus pettenkoferi*: L ≈ U > H *Streptococcus* spp.: L ≈ U > H
(41)	–	*Firmicutes*: H > L *Proteobacteria*: L > H	*Streptococcaceae*: L > H *Rhodobacteraceae*: L > H *Campylobacteraceae*: L > H *Staphylococcaceae*: H > L *Propionibacteriaceae*: H > L	*Paracoccus*: L > H	*Propionibacterium acnes*: H > L *Staphylococcus aureus*: H > L (p < 0.05)
(42)	α: H > U > L (p < 0.05) β: L > U > H (p < 0.05)	*Proteobacteria*: H > U > L	–	*Streptococcus*: L > U > H *Staphylococcus*: L > U > H	–
(43)	α: H > L (not significant) β: H > L	*Firmicutes*: H > L *Proteobacteria*: L > H (trunk p = 0.0113) *Actinobacteria*: H > L (p = 0.034)	–	*Streptococcus*: L > H *Staphylococcus*: H > L *Propionibacteria*: H > L (p = 0.061)	–
(44)	α: L > U >H (p < 0.001)	*Firmicutes*: L > U > H (p < 0.001) *Actinobacteria*: H > U > L (p < 0.01) *Proteobacteria*: H > L ≈ U (p < 0.001)	–	*Streptococcus*: L > U > H (p<0.001) *Propionibacterium*: H > U > L (p < 0.001)	*Propionibacterium acnes*: H > U > L (p < 0.001) *Staphylococcus aureus*: H > U (p < 0.001)

L, lesional skin from psoriasis; U, unaffected skin from psoriasis; H, skin from healthy control.

**Table 3:** Summary of published clinical trials involving skin fungus microbiota in psoriasis.

Study	Psoriasis Patients			Healthy Controls			Psoriasis type	Psoriasis severity	Sampling	Method	Diversity	Relative Abundance
	Number	Mean or Median Age	Sex ratio (M: F)	Number	Mean or Median Age	Sex ratio (M: F)						
(45)	12	63.8 ± 10.3 (53–78)	12: 0	12	59.3 ± 11.6 (55–75)	–	–	–	Scales collection	26S rRNA (D1 and D2)	α: L > H (p < 0.05)	*Filamentous fungi*: L > H *Malassezia*: H > L *M. restricta*: L > H (p < 0.05) *M. globosa*: H > L *Non-Malassezia yeast*: L > H
(46)	6	35.8 ± 9.2 (23–50)	2: 4	6	42.8 ± 18.9 (27–70)	3:3	–	PASI: 7.6 ± 2.6	Swab	26S rRNA (D1 and D2)	–	*Malassezia*: H > L
(47)	50	39 (9–76)	28: 22	50	–	–	Psoriasis vulgaris Palmoplantar psoriasis Psoriatic erythroderma	BSA: <3%: 3 subjects 3%–10%: 18 subjects >10%: 29 subjects	Scotch tape	26S rRNA	–	*Malassezia*: L > U > H (not significant) (Scalp: L > U, p = 0.03) *M. japonica*: BSA 3%–10% *M. globose*: BSA 10%–20% *M. slooffiae*: BSA 10%–20%
(48)	100	40.47 ± 11.03 (12–72)	44: 56	50	39.90 ± 11.45 (13–63)	22:28	Psoriasis vulgaris	–	Swab Biopsy	26S rRNA (D1 and D2)	–	*Candida*: L > U > H
(49)	2	33 (F), 58 (M)	1: 1	–	–	–	Mild psoriasis	–	Swab	5.8S rRNA	α: Not significant	Not significant
(34)	3	47.7 ± 11.8 (34–55)	3: 0	5	35.2 ± 11.3 (21–54)	2:3	–	–	Swab	18S rRNA 5.8S rRNA	α: Not significant (p = 0.78)	Not significant

PASI, Psoriasis Area and Severity Index; BSA, Body Surface Area score; PGA, Psoriasis Global Assessment score; L, lesional skin from psoriasis; U, unaffected skin from psoriasis; H, skin from healthy control.

#### 1.3.1 Bacteria

Cutaneotype 2, which is enriched for Firmicutes and Actinobacteria, is most prevalent in psoriatic subjects (43, 51). While in the trial of Yerushalmi et al. (37), it displayed a higher relative abundance of Firmicutes and lower relative abundance of Actinobacteria, with decreased alpha diversity, more heterogeneity, and reduced stability (**Tables 1**, **2**).

##### 1.3.1.1 Streptococcus

At the genus level, *Streptococcus* is the most common flora identified in psoriatic skin. Some people developed psoriasis outbreaks following *Streptococcus* infection. Evidence indicates that throat and nasal streptococcal infection, especially beta-hemolytic *Streptococcus pyogenes*, is responsible for guttate psoriasis (GP) and chronic plaque psoriasis (CPP). Tonsillectomy is proven to be a feasible strategy for *Streptococcus*-associated psoriasis, owing to a high frequency of cutaneous lymphocyte-associated antigen (CLA) + tonsil T cells that preferentially express IL-23 receptors, and these cells are postulated to be correlated with Th17, Th22, or Th1 polarization.

Superantigens are powerful T lymphocyte-stimulating agents. Streptococcal peptidoglycan (PG) has been implicated to function as superantigens, binding to class II major histocompatibility complex (MHC) molecules and Vβ segments of the T-cell receptor to initiate pathological responses and cytokine release in an antigen-specific manner (52). Similarly, streptococcal M proteins and pyrogenic exotoxin A, B, C also act as superantigens, binding directly to HLA-DR molecules on DCs, macrophages, and keratinocytes and activating T-lymphocyte subpopulations that express specific inflammation-associated Vβ families. It has been testified that selective aggregation of Vβ2+ T cells (p < 0.05) can be found in skin biopsies from all patients with GP lesions (53).

After recognizing IL-12 and superantigens that resemble M protein and keratin homologous peptides, CLA+ T cells could migrate to skin and subsequently react with streptococcal epitopes or skin-specific epitopes *via* molecular mimicry, resulting in psoriatic inflammation. In addition, CLA+ T cells are detected with decreased levels as psoriasis is ameliorated (54).

A preliminary study has demonstrated that dermal Th1 cell populations in GP and CPP lesions can selectively recognize lower MWt proteins (approximately 20–100 kDa) extracted from group A streptococci (GAS) cell wall, thereby increasingly producing IFN-γ in a self-HLA-DR allele-restricted manner. However, the contents of MWt proteins warrant further research to identify, implying that large proportions of Th1 cells specifically target streptococcal PG to initiate or exacerbate psoriatic inflammation (55). To sum up, psoriatic dermal streptococcal-specific CD4+ T-cell lines proliferate and release IFN-γ, IL-1, etc., in response to streptococcal PG (56). The theory of superantigen-activating T cells provides an explanation for 70% of GP patients who develop CPP (51).

##### 1.3.1.2 Staphylococcus

A majority of studies have manifested elevated levels of *Staphylococcus* on skin of psoriatic subjects. According to a meta-analysis involving 21 eligible studies, the presence of *Staphylococcus aureus* colonization in psoriatic patients is approximately 4.5 times higher than that of healthy controls (57). In the trial of Balci et al. (58), *S. aureus* was cultivated from lesional skin in approximately 64% of psoriatic patients, significantly higher than about 30% from non-lesional and healthy control samples, and 60% of patients were detected with Staphylococcus enterotoxins (*se*) and toxic shock syndrome toxin-1 (*TSST-1*). Genes encoding *sea*, *seb*, *sec*, *sed*, Panton–Valentine leukocidin (*PVL*), exfoliative toxin b (*etb*), *TSST-1*, and their carried accessory gene regulatory (agr) locus that regulates protease secretion and promotes *S. aureus* aggregation to the skin probably act as superantigen for psoriatic attack (59). Keratinocyte expression of HLA-DR that indirectly acting as a mediator binds superantigens, along with its secreted TNF concurrently trigger inflammatory cascades. In the murine experiment performed by Chang et al. (39), strong Th17 polarization was detected in mice colonized with *S. aureus* while absent in those with *Staphylococcus epidermidis* or un-colonized controls. Moreover, high PASI scores are significantly correlated with toxin-positive *S. aureus* colonization (58, 60). Similar to *Streptococcus*, staphylococcal PG could also be recognized by psoriatic T-cell lines through IFN-γ (56).

Paradoxically, in the trial of Elfatoiki et al. (61), *S. aureus* colonization was significantly lower in the lesional psoriatic skin than in controls (3% *vs.* 27.3%) and indicated that *Staphylococcus* may not be indispensable in provoking psoriasis.

##### 1.3.1.3 Other Bacterial Communities

Consistent with most clinical trials, Yan et al. (62) reported a decreased level of *Propionibacterium* in psoriasis lesions compared to controls. *Propionibacterium* can produce propionate and radical oxygenase (RoxP) that reduce oxidative stress and prevent skin inflammation (59). And its immunomodulatory constituents could protect skin barrier against external aggression (44). Hence, the underrepresentation of *Propionibacterium* may confer incapability to regulate the balance in the oxidant–antioxidant system, resulting in a disordered redox homeostasis. Moreover, *Propionibacterium acnes* strains have been demonstrated to differentially modulate Th17 cells of varied phenotypes in the presence of IL-2 and IL-23 to maintain homeostasis (63).

*Corynebacterium* is displayed with decreased abundance in trials. Plasmacytoid dendritic cells (pDCs) may have a direct pathological role through IFN production in psoriasis (64). It is speculated that *Corynebacterium* possesses an anti-inflammatory capability for negatively regulating “interferon signaling,” thus, its reduction causes a higher propensity to develop psoriasis onset or exacerbation (50).

With preclinical reports indicating that *Rhodobacter* has the anti-inflammatory capability to produce lycogen to prevent procollagen downregulation and inhibit NF-κB pathway, its decreased abundance is speculated to influence skin barrier and be involved in the pathogenesis of psoriasis (41).

#### 1.3.2 Fungus

Antifungal agents have yielded great efficacy in scalp psoriasis, suggesting the potential role of skin fungus in provocation or deterioration of psoriasis. Imiquimod (IMQ), a TLR7 agonist, has been applied to promote psoriasis-like skin inflammation *via* the IL-23/IL-17 axis in murine experiments (65). Hurabielle et al. (66) compared two experimental psoriatic models respectively associated with fungus/IMQ (*Candida albicans*, *Malassezia furfur*, and *Trichophyton mentagrophytes*) and IMQ alone, with the findings suggesting that fungal preexposure could significantly allow a better psoriatic model, depending on Th17 responses and neutrophil extracellular traps (NETs) (**Table 3**).

##### 1.3.2.1 Malassezia

Patch testing with inactivated *Malassezia* has been demonstrated to be capable of inducing clinically and histologically psoriasis-like lesions (67). *Malassezia* produces lipases and phospholipases that impair epidermal barrier, chemoattractants for polymorphonuclear leukocytes, as well as cross-reactive allergens leading to sensitization. In the study of Rudramurthy et al. (47), *M. furfur* is the predominant species (70.6%). *Malassezia* yeasts could do damage to the skin and stress predisposed keratinocytes then secondarily amplify AMPs. On the other hand, the study of Baroni et al. (68) showed a TLR2-dependence of IL-8 and HBD-2 in *M. furfur*-treated keratinocytes. Additionally, TLR2 can pair with TLR1 or TLR6, thus leading to large amounts of cytokine induction and ultimately chronic systemic inflammation in psoriasis (68).

Higher serum levels of IgG and significantly lower IgA against *M. furfur* were reported in patients developing psoriasis vulgaris. Antibodies to some constituents of *Malassezia* are also found in psoriatic lesions. It is also suggested that *M. furfur* can upregulate the expression of transforming growth factor (TGF)-β1, integrin, and heat shock protein 70 (HSP70) in an AP-1-dependent manner in human keratinocytes, thereby modulating cell cycle acceleration and favoring the exacerbation of psoriasis (69).

Previous studies showed that *M. furfur* could invade skin barrier and modulate immunomodulatory cytokine synthesis by downregulating IL-1α and by inhibiting IL-6 and TNF-α and by upregulating IL-10 and TGF-β1, thereby enhancing inflammatory responses (70).

##### 1.3.2.2 Candida

A meta-analysis revealed that *Candida* possesses significantly higher detection rates on mucosal membranes in psoriasis compared with controls (71). Another evaluation manifested that *Candida* could be isolated from 15% of psoriatic patients while 4% in controls. The serum IgM, IgG, and IgA against *C. albicans* were also prominently less in patients (p < 0.05), whereas no definite evidence has demonstrated the association between serum antibodies and its colonization with PASI (p > 0.05) (48). Whereas recent study holds that *Candida* colonization is positively associated with PASI (72).

In a murine experiment, neutrophil infiltration is notably observed in groups with preexposure to *C. albicans*. It is proposed that driven by candidal colonization, polarized IL-17-producing T cells including Th17, Tc17, and γδ T cells accumulate to concurrently promote IMQ pathology and exacerbate psoriasis-like skin inflammation in a dectin-1/Langerhans cell-dependent manner (66). In response to the skin barrier disruption, LCs recognize *C. albicans* yeasts through the C-type lectin receptor (CLR) dectin-1 and induce Th17 immune responses in the context of IL-6 (69, 70). *C. albicans* would stimulate nociceptors and could induce the neuropeptide calcitonin gene-related peptide (CGRP) *via* CLR dectin-1, and as a result drive dermal DCs and T cells to produce IL-23 and IL-17, which may lead to the occurrence of psoriasis (73, 74). The study found that in mice deficient in LCs, *C. albicans* is incapable of promoting neutrophil recruitment and IMQ-induced Th17 cell accumulation. Similarly, in mice deficient in dectin-1 or the gene encoding its downstream adaptor molecule CARD9, enhanced inflammation associated with preexposure to *C. albicans* was also abolished. Therefore, a Langerhans cell/dectin-1 axis plays a central role for the activated Th17 pathway in *C. albicans*/IMQ-treated mice (66).

It is also acknowledged that *C. albicans*-associated experimental psoriasis is characterized by enhanced neutrophil response and NET-mediated pathology. NET, the aberrant neutrophil activation/death, could be triggered by skin microorganisms through various downstream effector proteins (75). In the absence of NETosis inhibitor, psoriatic symptoms are exacerbated (76). NETs activate pDC accumulation to sense microbial DNA *via* TLR9 and TLR7 signaling, thus promoting type I IFN expression and may constitute a fundamental trigger of autoimmune pathology (77); in this process, LL-37 also potentiates TLR9 activation (78). In the clinical trial conducted by Hu et al. (79), NET parameters were positively correlated with PASI.

Nakajima et al. (80) also found that in murine models, *C. albicans* topical association significantly enhanced skin inflammation *via* promoting IL-17A production from CD4+ effector T cells in the skin and lymph nodes. It is also indicated that the surface proteins of *C. albicans* may act like superantigens and trigger Th17 activation through pro-inflammatory cytokines especially IL-23, which are essential for host defense against *C. albicans*. Additionally, IL-17 recruits neutrophils to resist *Candida* through direct phagocytosis, NET formation, and a substantial number of AMPs (81). Th9 cell populations, which produce large amounts of IL-9, increase in psoriatic skin lesions and connect the innate and adaptive immune system against *C. albicans* infection (82). In *C. albicans*/IMQ-treated samples, functional analysis manifested more significant enrichment for cytokine–cytokine receptor interaction along with positively regulatory genes associated with MAPK cascade, which could be seen enriched in human psoriasis as well.

#### 1.3.3 Virus

The role of virus in psoriasis is more obscure. PDCs infiltrate inflammatory psoriatic skin and produce large amounts of IFN-α on viral stimulation *via* TLR-7 and TLR-9, subsequently triggering T-cell cascade to potentiate Th1 cell bias and initiate psoriasis (20).

Previous cohort studies have revealed that people obtaining human papillomavirus (HPV) infection exhibited a 1.177 times greater risk of subsequently developing psoriasis particularly inverse psoriasis than did those in the healthy population, in which age acted as a prominent modifier (83). In the trial of Favre et al. HPV DNA was detected in 91.7% of 48 psoriatic skin samples. HPV5, HPV36, and HPV1 were 89.4%, 84.2%, and 42.1%, respectively, disclosing that psoriasis may be a reservoir for HPV5 (84). Simeone et al. (85) performed a clinical trial on 11 psoriatic patients, with the results showing the presence of HPV5 in 64% psoriatic keratinocytes, and concluded that viral replication in the psoriatic keratinocytes may trigger epidermal hyperproliferation along with antigen stimulation and lead to an autoimmune cascade. In hairs of psoriatic patients, the most prevalent HPV type in all tested samples was HPV-38, followed by HPV-25 (86).

With regard to psoriatic patients infected with hepatitis C virus (HCV), Chun et al. (81) found significantly higher LL37, TLR9, and IFN-y expression in lesional skin. It has been proposed that the cutaneous load of HCV infection is positively associated with PASI *via* detecting HCV protein and RNA expression in serum (82, 87).

For another, Teng et al. (88) proposed that the inverted CD4+/CD8+ ratio could induce keratinocytes aberrantly to express HLA-DR through IFN-γproduction in human immunodeficiency virus (HIV)-associated psoriasis.

#### 1.3.4 Other Microorganisms

In addition to the aforementioned skin microbiota, evolving knowledge indicates that there is still a wide range of microorganisms predisposing patients to developing psoriasis, such as *Helicobacter pylori*, *Porphyromonas gingivalis*, *Chlamydiae*, coronavirus disease 2019 (COVID-19), which presumably function as superantigens to initiate T-cell responses (89).

## 2 Discussion

Given the concern that the efficacy of off-the-shelf treatment products for psoriasis is limited hitherto, fully understanding the pathogenesis of psoriasis remains a priority for future research. Microbiome research is very prevalent at present. Whether the skin dysbiosis consists in the primary etiological significance or is secondary to psoriasis (or both) has yet to be completely characterized. Moreover, no consensus microorganisms have been directly identified. The generally accepted viewpoint holds that the altered microbiota probably serves potentially as a trigger or exacerbator for psoriasis. This review summarizes the outcome of multiple preceding clinical trials relevant to the altered skin microbiome in psoriatic patients, with a primary focus on mechanisms underlying how they interplay with the host immune system.

The field of the psoriatic skin microbiome is relatively new, and there are few studies on the specific and novel therapies targeting skin microbiome, thus leaving us considerable room to excavate. The manipulation of skin microbiome for treatment efficacy that preliminary research mentioned mostly refers to the conventional therapeutic options such as topical or systemic antibiotics that reduce susceptible bacterial species. Additionally, narrow-band ultraviolet radiation (NB-UVB) and balneotherapy have been demonstrated to induce an alteration of lesional skin microbiota and the improvement of psoriatic progression (90, 91). On the other hand, there is a direct link between gut and skin microbiota (92). Human skin microbiome composition can also be modulated through manipulated therapy of the gut microbiome (93). Researchers hold that probiotics may exert immunomodulatory effects on skin and can strengthen its barrier function against hazardous flora (94). Navarro-López et al. (95) had performed probiotic therapy on 80 psoriatic patients, with PASI75 reaching 66.7% and lower risk of relapse during follow-up compared to placebo group. Moreover, skin microbiome transplantation to diseased skin has yet to be regarded as another possible approach (96). Given the aforementioned encouraging efficacy on blocking disease progression, it is expected that more skin microbiome-related intervention to alleviate or cure this dermatosis would be developed in future research. For example, transdermal drug delivery must produce a milder side effect profile than current systemic medications. Besides, complement therapeutics are under investigation to potentially modulate the skin microbiota and even treat psoriasis (97).

Unlike atopic dermatitis, it is more than likely that the potential pathogens of psoriasis involve multiple species. Much about the potential role of viruses in psoriatic inflammation remains unclear. In future research, the upmost challenge might be to identify the extent to which skin microbiome plays a role in psoriatic pathogenesis and be modulated, and we should also identify one or several microbes that exert predominant influence on psoriasis, therefore enhancing the accuracy of modulation. Nevertheless, the heterogeneous parameters including genetics, diet, treatment exclusion, and taxonomic levels probably render the results reported in clinical trials less authentic and comparable. Furthermore, even in a single specific trial, the site- and microenvironment-based matching between lesion and control samples might be absent. As discussed, the preliminary studies only elaborate the correlative relationship between skin microbiome and psoriatic state as a whole. In the forthcoming decades, further experimentation to validate their definite association, and performed on isolated microbes, presents a principal direction. For one thing, a new set of standardized analysis protocol, including study population, sampling and processing methodology, would be a useful first step to interpret microbiome data. For instance, healthy people who have a common living background with psoriatic patients ought to be enrolled in a control group to reduce the bias. For another, in terms of taxonomic levels, it is critical to apply strain-level rather than species-level resolution approaches to unravel the microbial signatures associated with psoriasis; particularly, there are still vast unprofiled and undetermined regions about cutaneous viruses. It is proposed that gene transcriptomics is a suitable predictor of disease severity. Nevertheless, there is another tough problem regarding whether the sequence reads in skin swabs could map to specific functional genomes, thus making microorganisms classifiable (98). It still remains difficult to identify the specific driven genes. A substantial number of large-scale prospective longitudinal clinical trials and proof-of-concept experiments in murine models are also required to trace the dynamics of microbial populations during the onset and progression of psoriasis, which may give some enlightenment to predictive approaches for therapeutic responses through particular transcriptomic and microbial biomarkers with disease severity.

Notably, it is hypothesized that skin microbiome composition is associated with the development of psoriatic comorbidities. Initial studies show that its reduced diversity, which may weaken the skin protective function to trigger an immune response, might be a signature for psoriatic patients with a higher risk to develop PSA. It also showed that antibiotics have the potential to reduce the risk of comorbidities in psoriatic patients (96, 99). Whether the skin microbiota is involved in its comorbidity association remains to be further explored. It is expected to develop preventative measures to intervene to halt the progression of PSA.

Taken together, endeavors should be made in seeking out the deeper association between skin microbiome and developing microbiota-related interventions to mitigate psoriasis, such as selective modulation of isolated microbiota *via* intraindividual or interindividual skin microbiota transplantation, facilitating gut–skin cross-talk *via* prebiotics, and manipulating microbial pathways by targeting microbial metabolites through pharmacologic inhibitors, and hence, eventually improving clinical outcomes of psoriatic patients. In addition, the characteristics of chronic inflammation, along with its relevant metabolic comorbidity, concurrently indicate the significance of metabolomics in psoriasis. The extent to which skin microbiota play a role in the pathogenesis in psoriasis and which it can be modulated is certainly a critical consideration, and the potential function of skin microbiota on regulating global metabolism in psoriasis is a promising field. Hopefully, microbiome-specific targeted treatments or even curative paradigms for this dermatosis will come true in the upcoming period.

## 3 Conclusion

Psoriasis is mediated by immunological and external factors as a commonly seen chronic dermatosis in the population. Given the previous findings, the existing immunopathogenesis for psoriatic inflammation mainly involves IL-23/Th17 axis, along with large amounts of cytokines and immunocytes. Also, skin microbiota could regulate the cutaneous immune tolerance. In psoriatic skin, it is observed that the microorganism composition has altered, which interplays with host innate and adaptive immunity and potentially constitutes the pathogenesis of psoriasis. Some treatments regarding modifying the skin microbiome have been manifested to yield certain efficacy in psoriasis, whereas this field is relatively new and there is a series of challenges existing in future research. It is expected that this potential pathogenesis could be verified and that novel therapeutics targeting the skin microbiome could be figured out to improve the clinical outcome of psoriatic patients.

## Author Contributions

This study was conceived and designed by YC. Screening of papers and data extraction were performed by XL and CO. Writing of the first draft of the article was performed by XL, CO, JZ, and JL. Tables and figure were prepared by XL, CO, FZ, and YZ. All authors contributed to the article and approved the submitted version.

## Conflict of Interest

The authors declare that the research was conducted in the absence of any commercial or financial relationships that could be construed as a potential conflict of interest.

## Publisher’s Note

All claims expressed in this article are solely those of the authors and do not necessarily represent those of their affiliated organizations, or those of the publisher, the editors and the reviewers. Any product that may be evaluated in this article, or claim that may be made by its manufacturer, is not guaranteed or endorsed by the publisher.

## References

[1] Parisi R Symmons DP Griffiths CE Ashcroft DM Identification, Management of P. Global Epidemiology of Psoriasis: A Systematic Review of Incidence and Prevalence. J Invest Dermatol (2013) 133(2):377–85. doi: 10.1038/jid.2012.339 23014338

[2] Polak K Bergler-Czop B Szczepanek M Wojciechowska K Fratczak A Kiss N. Psoriasis and Gut Microbiome-Current State of Art. Int J Mol Sci (2021) 22(9):4529. doi: 10.3390/ijms22094529 33926088 PMC8123672

[3] Navarro-Lopez V Nunez-Delegido E Ruzafa-Costas B Sanchez-Pellicer P Aguera-Santos J Navarro-Moratalla L. Probiotics in the Therapeutic Arsenal of Dermatologists. Microorganisms (2021) 9(7):1513. doi: 10.3390/microorganisms9071513 34361948 PMC8303240

[4] Catinean A Neag MA Mitre AO Bocsan CI Buzoianu AD. Microbiota and Immune-Mediated Skin Diseases-an Overview. Microorganisms (2019) 7(9):279. doi: 10.3390/microorganisms7090279 PMC6781142 31438634

[5] Edslev SM Agner T Andersen PS. Skin Microbiome in Atopic Dermatitis. Acta Derm Venereol (2020) 100(12):adv00164. doi: 10.2340/00015555-3514 32419029 PMC9189751

[6] Li CX You ZX Lin YX Liu HY Su J. Skin Microbiome Differences Relate to the Grade of Acne Vulgaris. J Dermatol (2019) 46(9):787–90. doi: 10.1111/1346-8138.14952 31290561

[7] Ganju P Nagpal S Mohammed MH Nishal Kumar P Pandey R Natarajan VT. Microbial Community Profiling Shows Dysbiosis in the Lesional Skin of Vitiligo Subjects. Sci Rep (2016) 6:18761. doi: 10.1038/srep18761 26758568 PMC4725359

[8] Huang C Yi X Long H Zhang G Wu H Zhao M. Disordered Cutaneous Microbiota in Systemic Lupus Erythematosus. J Autoimmun (2020) 108:102391. doi: 10.1016/j.jaut.2019.102391 31883828

[9] Chen D He J Li J Zou Q Si J Guo Y. Microbiome and Metabolome Analyses Reveal Novel Interplay Between the Skin Microbiota and Plasma Metabolites in Psoriasis. Front Microbiol (2021) 12:643449. doi: 10.3389/fmicb.2021.643449 33796091 PMC8007969

[10] Eppinga H Konstantinov SR Peppelenbosch MP Thio HB. The Microbiome and Psoriatic Arthritis. Curr Rheumatol Rep (2014) 16(3):407. doi: 10.1007/s11926-013-0407-2 24474190

[11] Olejniczak-Staruch I Ciazynska M Sobolewska-Sztychny D Narbutt J Skibinska M Lesiak A. Alterations of the Skin and Gut Microbiome in Psoriasis and Psoriatic Arthritis. Int J Mol Sci (2021) 22(8):3998. doi: 10.3390/ijms22083998 33924414 PMC8069836

[12] Vlachos C Gaitanis G Katsanos KH Christodoulou DK Tsianos E Bassukas ID. Psoriasis and Inflammatory Bowel Disease: Links and Risks. Psoriasis (Auckl) (2016) 6:73–92. doi: 10.2147/PTT.S85194 29387596 PMC5683131

[13] Bunte K Beikler T. Th17 Cells and the IL-23/IL-17 Axis in the Pathogenesis of Periodontitis and Immune-Mediated Inflammatory Diseases. Int J Mol Sci (2019) 20(14):3394. doi: 10.3390/ijms20143394 PMC6679067 31295952

[14] Xiao S Zhang G Jiang C Liu X Wang X Li Y. Deciphering Gut Microbiota Dysbiosis and Corresponding Genetic and Metabolic Dysregulation in Psoriasis Patients Using Metagenomics Sequencing. Front Cell Infect Microbiol (2021) 11:605825. doi: 10.3389/fcimb.2021.605825 33869074 PMC8047475

[15] Ranjan R Rani A Metwally A McGee HS Perkins DL. Analysis of the Microbiome: Advantages of Whole Genome Shotgun Versus 16S Amplicon Sequencing. Biochem Biophys Res Commun (2016) 469(4):967–77. doi: 10.1016/j.bbrc.2015.12.083 PMC4830092 26718401

[16] Boehncke W-H Schön MP. Psoriasis. Lancet (2015) 386(9997):983–94. doi: 10.1016/s0140-6736(14)61909-7 26025581

[17] Cai Y Fleming C Yan J. New Insights of T Cells in the Pathogenesis of Psoriasis. Cell Mol Immunol (2012) 9(4):302–9. doi: 10.1038/cmi.2012.15 PMC4132586 22705915

[18] Girolomoni G Strohal R Puig L Bachelez H Barker J Boehncke WH. The Role of IL-23 and the IL-23/TH17 Immune Axis in the Pathogenesis and Treatment of Psoriasis. J Eur Acad Dermatol Venereol (2017) 31(10):1616–26. doi: 10.1111/jdv.14433 PMC5697699 28653490

[19] Bettelli E Oukka M Kuchroo VK. T(H)-17 Cells in the Circle of Immunity and Autoimmunity. Nat Immunol (2007) 8(4):345–50. doi: 10.1038/ni0407-345 17375096

[20] Nestle FO Conrad C Tun-Kyi A Homey B Gombert M Boyman O. Plasmacytoid Predendritic Cells Initiate Psoriasis Through Interferon-Alpha Production. J Exp Med (2005) 202(1):135–43. doi: 10.1084/jem.20050500 PMC2212894 15998792

[21] Lowes MA Suárez-Fariñas M Krueger JG. Immunology of Psoriasis. Annu Rev Immunol (2014) 32(1):227–55. doi: 10.1146/annurev-immunol-032713-120225 PMC4229247 24655295

[22] Buchau AS Gallo RL. Innate Immunity and Antimicrobial Defense Systems in Psoriasis. Clin Dermatol (2007) 25(6):616–24. doi: 10.1016/j.clindermatol.2007.08.016 PMC2699547 18021900

[23] Gutowska-Owsiak D Schaupp AL Salimi M Selvakumar TA McPherson T Taylor S. IL-17 Downregulates Filaggrin and Affects Keratinocyte Expression of Genes Associated With Cellular Adhesion. Exp Dermatol (2012) 21(2):104–10. doi: 10.1111/j.1600-0625.2011.01412.x 22229441

[24] Elias PM Arbiser J Brown BE Rossiter H Man M-Q Cerimele F. Epidermal Vascular Endothelial Growth Factor Production is Required for Permeability Barrier Homeostasis, Dermal Angiogenesis, and the Development of Epidermal Hyperplasia. Am J Pathol (2008) 173(3):689–99. doi: 10.2353/ajpath.2008.080088 PMC2527083 18688025

[25] Okayama Y. Oxidative Stress in Allergic and Inflammatory Skin Diseases. Curr Drug Targets Inflammation Allergy (2005) 4(4):517–9. doi: 10.2174/1568010054526386 16127829

[26] Singh TP Schon MP Wallbrecht K Gruber-Wackernagel A Wang XJ Wolf P. Involvement of IL-9 in Th17-Associated Inflammation and Angiogenesis of Psoriasis. PloS One (2013) 8(1):e51752. doi: 10.1371/journal.pone.0051752 23335955 PMC3546056

[27] Matsumoto R Dainichi T Tsuchiya S Nomura T Kitoh A Hayden MS. Epithelial TRAF6 Drives IL-17–Mediated Psoriatic Inflammation. JCI Insight (2018) 3(15):e121175. doi: 10.1172/jci.insight.121175 PMC6129131 30089718

[28] Grinberg-Bleyer Y Dainichi T Oh H Heise N Klein U Schmid RM. Cutting Edge: Nf-κb P65 and C-Rel Control Epidermal Development and Immune Homeostasis in the Skin. J Immunol (2015) 194(6):2472–6. doi: 10.4049/jimmunol.1402608 PMC4355158 25681334

[29] Costello EK Lauber CL Hamady M Fierer N Gordon JI Knight R. Bacterial Community Variation in Human Body Habitats Across Space and Time. Science (2009) 326(5960):1694–7. doi: 10.1126/science.1177486 PMC3602444 19892944

[30] Naik S Bouladoux N Linehan JL Han SJ Harrison OJ Wilhelm C. Commensal-Dendritic-Cell Interaction Specifies a Unique Protective Skin Immune Signature. Nature (2015) 520(7545):104–8. doi: 10.1038/nature14052 PMC4667810 25539086

[31] Integrative HMPRNC. The Integrative Human Microbiome Project. Nature (2019) 569(7758):641–8. doi: 10.1038/s41586-019-1238-8 PMC6784865 31142853

[32] Ahmed N Huse SM Ye Y Zhou Y Fodor AA. A Core Human Microbiome as Viewed Through 16S rRNA Sequence Clusters. PloS One (2012) 7(6):e34242. doi: 10.1371/journal.pone.0034242 22719824 PMC3374614

[33] Grice EA Kong HH Conlan S Deming CB Davis J Young AC. Topographical and Temporal Diversity of the Human Skin Microbiome. Science (2009) 324(5931):1190–2. doi: 10.1126/science.1171700 PMC2805064 19478181

[34] Paulino LC Tseng CH Strober BE Blaser MJ. Molecular Analysis of Fungal Microbiota in Samples From Healthy Human Skin and Psoriatic Lesions. J Clin Microbiol (2006) 44(8):2933–41. doi: 10.1128/JCM.00785-06 PMC1594634 16891514

[35] Dainichi T Kitoh A Otsuka A Nakajima S Nomura T Kaplan DH. The Epithelial Immune Microenvironment (EIME) in Atopic Dermatitis and Psoriasis. Nat Immunol (2018) 19(12):1286–98. doi: 10.1038/s41590-018-0256-2 30446754

[36] Langan EA Griffiths CEM Solbach W Knobloch JK Zillikens D Thaci D. The Role of the Microbiome in Psoriasis: Moving From Disease Description to Treatment Selection? Br J Dermatol (2018) 178(5):1020–7. doi: 10.1111/bjd.16081 29071712

[37] Yerushalmi M Elalouf O Anderson M Chandran V. The Skin Microbiome in Psoriatic Disease: A Systematic Review and Critical Appraisal. J Trans Autoimmun (2019) 2:100009. doi: 10.1016/j.jtauto.2019.100009 PMC7388378 32743498

[38] Fry L Baker BS Powles AV Fahlen A Engstrand L. Is Chronic Plaque Psoriasis Triggered by Microbiota in the Skin? Br J Dermatol (2013) 169(1):47–52. doi: 10.1111/bjd.12322 23521130

[39] Chang H-W Yan D Singh R Liu J Lu X Ucmak D. Alteration of the Cutaneous Microbiome in Psoriasis and Potential Role in Th17 Polarization. Microbiome (2018) 6(1):154. doi: 10.1186/s40168-018-0533-1 30185226 PMC6125946

[40] Yan D Chang H Singh R Lai K Afifi L Lu X. 633 Role of the Cutaneous Microbiome in the Pathogenesis of Psoriasis. J Invest Dermatol (2017) 137(5):S109. doi: 10.1016/j.jid.2017.02.655

[41] Drago L De Grandi R Altomare G Pigatto P Rossi O Toscano M. Skin Microbiota of First Cousins Affected by Psoriasis and Atopic Dermatitis. Clin Mol Allergy (2016) 14(1):2. doi: 10.1186/s12948-016-0038-z 26811697 PMC4724956

[42] Alekseyenko AV Perez-Perez GI De Souza A Strober B Gao Z Bihan M. Community Differentiation of the Cutaneous Microbiota in Psoriasis. Microbiome (2013) 1(1):31. doi: 10.1186/2049-2618-1-31 24451201 PMC4177411

[43] Fahlén A Engstrand L Baker BS Powles A Fry L. Comparison of Bacterial Microbiota in Skin Biopsies From Normal and Psoriatic Skin. Arch Dermatol Res (2011) 304(1):15–22. doi: 10.1007/s00403-011-1189-x 22065152

[44] Gao Z Tseng CH Strober BE Pei Z Blaser MJ. Substantial Alterations of the Cutaneous Bacterial Biota in Psoriatic Lesions. PloS One (2008) 3(7):e2719. doi: 10.1371/journal.pone.0002719 18648509 PMC2447873

[45] Takemoto A Cho O Morohoshi Y Sugita T Muto M. Molecular Characterization of the Skin Fungal Microbiome in Patients With Psoriasis. J Dermatol (2015) 42(2):166–70. doi: 10.1111/1346-8138.12739 25510344

[46] Jagielski T Rup E Ziółkowska A Roeske K Macura AB Bielecki J. Distribution of Malassezia Species on the Skin of Patients With Atopic Dermatitis, Psoriasis, and Healthy Volunteers Assessed by Conventional and Molecular Identification Methods. BMC Dermatol (2014) 14(3):3. doi: 10.1186/1471-5945-14-3 24602368 PMC3975586

[47] Rudramurthy SM Honnavar P Chakrabarti A Dogra S Singh P Handa S. Association Ofmalasseziaspecies With Psoriatic Lesions. Mycoses (2014) 57(8):483–8. doi: 10.1111/myc.12186 24655111

[48] Taheri Sarvtin M Shokohi T Hajheydari Z Yazdani J Hedayati MT. Evaluation of Candidal Colonization and Specific Humoral Responses Against Candida Albicans in Patients With Psoriasis. Int J Dermatol (2014) 53(12):e555–e60. doi: 10.1111/ijd.12562 25427068

[49] Paulino LC Chi-Hong T Blaser MJ. Analysis of Malassezia Microbiota in Healthy Superficial Human Skin and in Psoriatic Lesions by Multiplex Real-Time Pcr. FEMS Yeast Res (2010) 3):460–71. doi: 10.1111/j.1567-1364.2008.00359.x 18294199

[50] Fyhrquist N Muirhead G Prast-Nielsen S Jeanmougin M Olah P Skoog T. Microbe-Host Interplay in Atopic Dermatitis and Psoriasis. Nat Commun (2019) 10(1):4703. doi: 10.1038/s41467-019-12253-y 31619666 PMC6795799

[51] Macias ES Pereira FA Rietkerk W Safai B. Superantigens in Dermatology. J Am Acad Dermatol (2011) 64(3):455–72; quiz 73-4. doi: 10.1016/j.jaad.2010.03.044 21315950

[52] Leung D Travers JB Giorno R Norris DA Kotb M. Evidence for a Streptococcal Superantigen-Driven Process in Acute Guttate Psoriasis. J Clin Invest (1995) 99(5):2106. doi: 10.1172/JCI118263 PMC185858 7593594

[53] El Ferezli J Jenbazian L Rubeiz N Kibbi A-G Zaynoun S Abdelnoor AM. Streptococcussp. Andstaphylococcus Aureusisolates From Patients With Psoriasis Possess Genes That Code for Toxins (Superantigens): Clinical and Therapeutic Implications. Immunopharmacol Immunotoxicology (2008) 30(2):195–205. doi: 10.1080/08923970801946808 18569077

[54] Sigurdardottir SL Thorleifsdottir RH Valdimarsson H Johnston A. The Association of Sore Throat and Psoriasis Might be Explained by Histologically Distinctive Tonsils and Increased Expression of Skin-Homing Molecules by Tonsil T Cells. Clin Exp Immunol (2013) 174(1):139–51. doi: 10.1111/cei.12153 PMC3784221 23750651

[55] GÖÇMen JS ŞAhİNer N KoÇAk M Karahan ZC. PCR Investigation of Panton-Valentine Leukocidin, Enterotoxin, Exfoliative Toxin,and Agr Genes in Staphylococcus Aureus Strains Isolated From Psoriasis Patients*. Turkish J Med Sci (2015) 45:1345–52. doi: 10.3906/sag-1408-54 26775393

[56] Baker BS Laman JD Powles A van der Fits L Voerman JSA Melief MJ. Peptidoglycan and Peptidoglycan-Specific Th1 Cells in Psoriatic Skin Lesions. J Pathol (2006) 209(2):174–81. doi: 10.1002/path.1954 16493599

[57] Ng CY Huang YH Chu CF Wu TC Liu SH. Risks for Staphylococcus Aureus Colonization in Patients With Psoriasis: A Systematic Review and Meta-Analysis. Br J Dermatol (2017) 177(4):967–77. doi: 10.1111/bjd.15366 28160277

[58] Balci DD Duran N Ozer B Gunesacar R Onlen Y Yenin JZ. High Prevalence of Staphylococcus Aureus Cultivation and Superantigen Production in Patients With Psoriasis. Eur J Dermatol (2009) 19(3):238–42. doi: 10.1684/ejd.2009.0663 19286488

[59] Allhorn M Arve S Brüggemann H Lood R. A Novel Enzyme With Antioxidant Capacity Produced by the Ubiquitous Skin Colonizer Propionibacterium Acnes. Sci Rep (2016) 6(1):36412. doi: 10.1038/srep36412 27805044 PMC5090349

[60] Tomi NS Kranke B Aberer E. Staphylococcal Toxins in Patients With Psoriasis, Atopic Dermatitis, and Erythroderma, and in Healthy Control Subjects. J Am Acad Dermatol (2005) 53(1):67–72. doi: 10.1016/j.jaad.2005.02.034 15965423

[61] Elfatoiki FZ El Azhari M El Kettani A Serhier Z Othmani MB Timinouni M. Psoriasis and Staphylococcus Aureus Skin Colonization in Moroccan Patients. Pan Afr Med J (2016) 23:33. doi: 10.11604/pamj.2016.23.33.7198 27200138 PMC4856496

[62] Yan D Issa N Afifi L Jeon C Chang HW Liao W. The Role of the Skin and Gut Microbiome in Psoriatic Disease. Curr Dermatol Rep (2017) 6(2):94–103. doi: 10.1007/s13671-017-0178-5 28804689 PMC5552074

[63] Agak GW Kao S Ouyang K Qin M Moon D Butt A. Phenotype and Antimicrobial Activity of Th17 Cells Induced by Propionibacterium Acnes Strains Associated With Healthy and Acne Skin. J Invest Dermatol (2018) 138(2):316–24. doi: 10.1016/j.jid.2017.07.842 PMC5794628 28864077

[64] Reizis B Colonna M Trinchieri G Barrat F Gilliet M. Plasmacytoid Dendritic Cells: One-Trick Ponies or Workhorses of the Immune System? Nat Rev Immunol (2011) 11(8):558–65. doi: 10.1038/nri3027 PMC4157822 21779033

[65] van der Fits L Mourits S Voerman JS Kant M Boon L Laman JD. Imiquimod-Induced Psoriasis-Like Skin Inflammation in Mice is Mediated *via* the IL-23/IL-17 Axis. J Immunol (2009) 182(9):5836–45. doi: 10.4049/jimmunol.0802999 19380832

[66] Hurabielle C Link VM Bouladoux N Han SJ Merrill ED Lightfoot YL. Immunity to Commensal Skin Fungi Promotes Psoriasiform Skin Inflammation. Proc Natl Acad Sci USA (2020) 117(28):16465–74. doi: 10.1073/pnas.2003022117 PMC7368261 32601220

[67] Lober CW Belew PW Rosenberg EW Bale G. Patch Tests With Killed Sonicated Microflora in Patients With Psoriasis. Arch Dermatol (1982) 118(5):322–5. doi: 10.1001/archderm.1982.01650170036019 6211147

[68] Baroni A Orlando M Donnarumma G Farro P Iovene MR Tufano MA. Toll-Like Receptor 2 (TLR2) Mediates Intracellular Signalling in Human Keratinocytes in Response to Malassezia Furfur. Arch Dermatol Res (2006) 297(7):280–8. doi: 10.1007/s00403-005-0594-4 16283346

[69] Kashem SW Igyarto BZ Gerami-Nejad M Kumamoto Y Mohammed JA Jarrett E. Candida Albicans Morphology and Dendritic Cell Subsets Determine T Helper Cell Differentiation. Immunity (2015) 42(2):356–66. doi: 10.1016/j.immuni.2015.01.008 PMC4343045 25680275

[70] Doebel T Voisin B Nagao K. Langerhans Cells - the Macrophage in Dendritic Cell Clothing. Trends Immunol (2017) 38(11):817–28. doi: 10.1016/j.it.2017.06.008 28720426

[71] Pietrzak A Grywalska E Socha M Rolinski J Franciszkiewicz-Pietrzak K Rudnicka L. Prevalence and Possible Role of Candida Species in Patients With Psoriasis: A Systematic Review and Meta-Analysis. Mediators Inflamm (2018) 2018:9602362. doi: 10.1155/2018/9602362 29853795 PMC5960518

[72] Ovčina-Kurtović N Kasumagić-Halilović E Helppikangans H Begić J. Prevalence of Candida Species in Patients With Psoriasis. Acta dermatovenerologica Croatica (2016) 24(3):209–13. 27663922

[73] Kashem Sakeen W Riedl Maureen S Yao C Honda Christopher N Vulchanova L Kaplan Daniel H. Nociceptive Sensory Fibers Drive Interleukin-23 Production From CD301b+ Dermal Dendritic Cells and Drive Protective Cutaneous Immunity. Immunity (2015) 43(3):515–26. doi: 10.1016/j.immuni.2015.08.016 PMC4607048 26377898

[74] Maruyama K Takayama Y Kondo T Ishibashi KI Sahoo BR Kanemaru H. Nociceptors Boost the Resolution of Fungal Osteoinflammation *via* the TRP Channel-CGRP-Jdp2 Axis. Cell Rep (2017) 19(13):2730–42. doi: 10.1016/j.celrep.2017.06.002 28658621

[75] Papayannopoulos V. Neutrophil Extracellular Traps in Immunity and Disease. Nat Rev Immunol (2018) 18(2):134–47. doi: 10.1038/nri.2017.105 28990587

[76] Zabieglo K Majewski P Majchrzak-Gorecka M Wlodarczyk A Grygier B Zegar A. The Inhibitory Effect of Secretory Leukocyte Protease Inhibitor (SLPI) on Formation of Neutrophil Extracellular Traps. J Leukoc Biol (2015) 98(1):99–106. doi: 10.1189/jlb.4AB1114-543R 25917460 PMC4467168

[77] Guiducci C Tripodo C Gong M Sangaletti S Colombo MP Coffman RL. Autoimmune Skin Inflammation is Dependent on Plasmacytoid Dendritic Cell Activation by Nucleic Acids *via* TLR7 and TLR9. J Exp Med (2010) 207(13):2931–42. doi: 10.1084/jem.20101048 PMC3005224 21115693

[78] Lande R Gregorio J Facchinetti V Chatterjee B Wang YH Homey B. Plasmacytoid Dendritic Cells Sense Self-DNA Coupled With Antimicrobial Peptide. Nature (2007) 449(7162):564–9. doi: 10.1038/nature06116 17873860

[79] Hu SC-S Yu H-S Yen F-L Lin C-L Chen G-S Lan C-CE. Neutrophil Extracellular Trap Formation is Increased in Psoriasis and Induces Human β-Defensin-2 Production in Epidermal Keratinocytes. Sci Rep (2016) 6(1):31119. doi: 10.1038/srep31119 27493143 PMC4974609

[80] Nakajima S Harrison O Merrill E Linehan J Belkaid Y. 648 Candida Albicans Colonization Exacerbates Skin Inflammation in a Murine Model of Psoriasis. J Invest Dermatol (2017) 137(5):S112. doi: 10.1016/j.jid.2017.02.670

[81] Chun K Afshar M Audish D Kabigting F Paik A Gallo R. Hepatitis C may Enhance Key Amplifiers of Psoriasis. J Eur Acad Dermatol Venereol (2017) 31(4):672–8. doi: 10.1111/jdv.13578 27184185

[82] Farag AGA Elshayb EE Sharaky DRA Elashafey EN Khadra AAEA. Role of HCV Infection in Psoriasis: A Clinical and Immunohistochemical Study. J Clin Diagn Res (2019) 13(5):WC01–6. doi: 10.7860/jcdr/2019/39627.12833

[83] Chen M-L Kao W-M Huang J-Y Hung Y-M Wei JC-C. Human Papillomavirus Infection Associated With Increased Risk of New-Onset Psoriasis: A Nationwide Population-Based Cohort Study. Int J Epidemiol (2020) 49(3):786–97. doi: 10.1093/ije/dyaa027 32176290

[84] Stephens GL Swerdlow B Benjamin E Coyle AJ Humbles A Kolbeck R. IL-9 is a Th17-Derived Cytokine That Limits Pathogenic Activity in Organ-Specific Autoimmune Disease. Eur J Immunol (2011) 41(4):952–62. doi: 10.1002/eji.201040879 21360526

[85] Simeone P Teson M Latini A Carducci M Venuti A. Human Papillomavirus Type 5 in Primary Keratinocytes From Psoriatic Skin. Exp Dermatol (2005) 14(11):824–9. doi: 10.1111/j.1600-0625.2005.00358.x 16232304

[86] Wolf P Seidl H Back B Binder B Hofler G Quehenberger F. Increased Prevalence of Human Papillomavirus in Hairs Plucked From Patients With Psoriasis Treated With Psoralen-UV-a. Arch Dermatol (2004) 140(3):317–24. doi: 10.1001/archderm.140.3.317 15023775

[87] Gabr SA Berika MY Alghadir AH. Apoptosis and Clinical Severity in Patients With Psoriasis and HCV Infection. Indian J Dermatol (2014) 59(3):230–6. doi: 10.4103/0019-5154.131377 PMC4037940 24891650

[88] Teng Y Xie W Tao X Liu N Yu Y Huang Y. Infection-Provoked Psoriasis: Induced or Aggravated (Review). Exp Ther Med (2021) 21(6):567. doi: 10.3892/etm.2021.9999 33850539 PMC8027725

[89] Rademaker M Agnew K Anagnostou N Andrews M Armour K Baker C. Psoriasis and Infection. A Clinical Practice Narrative. Australas J Dermatol (2019) 60(2):91–8. doi: 10.1111/ajd.12895 30079566

[90] Assarsson M Duvetorp A Dienus O Soderman J Seifert O. Significant Changes in the Skin Microbiome in Patients With Chronic Plaque Psoriasis After Treatment With Narrowband Ultraviolet B. Acta Derm Venereol (2018) 98(4):428–36. doi: 10.2340/00015555-2859 29199351

[91] Sophie S Christian O. Skin Microbiome in Patients With Psoriasis Before and After Balneotherapy at the Thermal Care Center of La Roche-Posay. J Am Acad Dermatol (2016) 74(5):AB276. doi: 10.1016/j.jaad.2016.02.1062 26659932

[92] Salem I Ramser A Isham N Ghannoum MA. The Gut Microbiome as a Major Regulator of the Gut-Skin Axis. Front Microbiol (2018) 9:1459. doi: 10.3389/fmicb.2018.01459 30042740 PMC6048199

[93] Paetzold B Willis JR Pereira de Lima J Knodlseder N Bruggemann H Quist SR. Skin Microbiome Modulation Induced by Probiotic Solutions. Microbiome (2019) 7(1):95. doi: 10.1186/s40168-019-0709-3 31234928 PMC6591853

[94] Benhadou F Mintoff D Schnebert B Thio HB. Psoriasis and Microbiota: A Systematic Review. Diseases (2018) 6(2):47. doi: 10.3390/diseases6020047 PMC6023392 29865237

[95] Navarro-Lopez V Martinez-Andres A Ramirez-Bosca A Ruzafa-Costas B Nunez-Delegido E Carrion-Gutierrez MA. Efficacy and Safety of Oral Administration of a Mixture of Probiotic Strains in Patients With Psoriasis: A Randomized Controlled Clinical Trial. Acta Derm Venereol (2019) 99(12):1078–84. doi: 10.2340/00015555-3305 31453631

[96] Thio HB. The Microbiome in Psoriasis and Psoriatic Arthritis: The Skin Perspective. J Rheumatol Suppl (2018) 94:30–1. doi: 10.3899/jrheum.180133 29858350

[97] Chehoud C Rafail S Tyldsley AS Seykora JT Lambris JD Grice EA. Complement Modulates the Cutaneous Microbiome and Inflammatory Milieu. Proc Natl Acad Sci USA (2013) 110(37):15061–6. doi: 10.1073/pnas.1307855110 PMC3773768 23980152

[98] Oh J Byrd AL Deming C Conlan S Program NCS Kong HH. Biogeography and Individuality Shape Function in the Human Skin Metagenome. Nature (2014) 514(7520):59–64. doi: 10.1038/nature13786 25279917 PMC4185404

[99] Wilson FC Icen M Crowson CS McEvoy MT Gabriel SE Kremers HM. Incidence and Clinical Predictors of Psoriatic Arthritis in Patients With Psoriasis: A Population-Based Study. Arthritis Rheum (2009) 61(2):233–9. doi: 10.1002/art.24172 PMC3061343 19177544

